# Psychosocial Characteristics and Social Networks of Suicidal Prisoners: Towards a Model of Suicidal Behaviour in Detention

**DOI:** 10.1371/journal.pone.0068944

**Published:** 2013-07-29

**Authors:** Adrienne Rivlin, Keith Hawton, Lisa Marzano, Seena Fazel

**Affiliations:** 1 Centre for Suicide Research, University Department of Psychiatry, Warneford Hospital, Oxford, United Kingdom; 2 Psychology Department, Middlesex University, The Burroughs, London, United Kingdom; Catholic University of Sacred Heart of Rome, Italy

## Abstract

Prisoners are at increased risk of suicide. Investigation of both individual and environmental risk factors may assist in developing suicide prevention policies for prisoners and other high-risk populations. We conducted a matched case-control interview study with 60 male prisoners who had made near-lethal suicide attempts in prison (cases) and 60 male prisoners who had not (controls). We compared levels of depression, hopelessness, self-esteem, impulsivity, aggression, hostility, childhood abuse, life events (including events occurring in prison), social support, and social networks in univariate and multivariate models. A range of psychosocial factors was associated with near-lethal self-harm in prisoners. Compared with controls, cases reported higher levels of depression, hopelessness, impulsivity, and aggression, and lower levels of self-esteem and social support (all p values <0.001). Adverse life events and criminal history factors were also associated with near-lethal self-harm, especially having a prior prison spell and having been bullied in prison, both of which remained significant in multivariate analyses. The findings support a model of suicidal behaviour in prisoners that incorporates imported vulnerability factors, clinical factors, and prison experiences, and underscores their interaction. Strategies to reduce self-harm and suicide in prisoners should include attention to such factors.

## Introduction

Rates of suicide in male prisoners in many high income countries are around three to seven times higher than in the general population [1]. Therefore, several national suicide prevention strategies specifically target this high risk group [2], [3]. Such strategies would benefit from more information on specific prisoner subgroups with different risk profiles [4], and a deeper understanding of psychosocial factors, particularly those that are not routinely collected by prison administrations.

Theoretical models of suicide suggest that suicidal behaviour is rarely the result of a single cause or event, but rather depends on the cumulative and interactive effects of several social, environmental, familial, personality and mental health factors [5], [6], [7]. These are likely to involve an underlying vulnerability to suicide (mostly defined in terms of biological and psychological traits), which becomes heightened under the influence of particular stressors [8]. In a prison setting, these may include general aspects of the regime, such as adjustment to the prison situation, loss of freedom, and removal from a familiar environment [9], as well as more specific aspects of prison life, including a lack of purposeful activity (i.e. access to activities to keep occupied such as work or education) [10], withdrawal from drugs or alcohol [4], receiving bad news [11], being in a single cell or in segregation [12], violence and victimization [13], and boredom [14].

A prisoner’s vulnerability to these factors may in turn be influenced by personal characteristics and predispositions that are ‘imported’ into the prison. Among them are current and lifetime psychopathology [15], physical illness [16], adverse life events, such as a history of childhood trauma, and personality characteristics likely to influence an individual’s opinion of themselves, perceptions of and adaptations to the environment, and the likelihood of acting on suicidal feelings [17], [18].

Consistent with this life-course model of the aetiology of suicide, there is growing awareness of the need to investigate a wide range of both individual and environmental factors in order to better understand and reduce the incidence of suicidal behaviour in prisons. Yet much of the research in this area has focused on a relatively narrow range of variables [12]. An important exception is a study in the UK by the Office for National Statistics (ONS) [19], in which demographic, social and psychiatric correlates of suicidal behaviour in prisons were explored in a large sample of male and female prisoners. However, this study did not include direct assessment of psychological states or traits, and examined as an outcome lifetime and previous suicidality (based on self-reported intent), rather than suicidal behaviour occurring exclusively during incarceration. Furthermore, the ONS study focused on the broad categories of suicidal ideation and attempts as proxies for suicide. However, there is evidence that physically dangerous and medically severe self-harm acts provide a better approximation of actual suicide than other forms of self-harming behaviour or suicide attempts [20], [21]. In addition, near-lethal acts of self-harm are an important and prevalent problem in their own right in prisons, for which targeted interventions should be considered [4].

We studied male prisoners who had made near-lethal suicide attempts in prison and compared them with prisoners who had not engaged in near-lethal self-harm in custody. We had two main research questions. The first was how do psychological characteristics, namely depressive symptoms, hopelessness, self-esteem, impulsivity, aggression and hostility, all of which have been implicated in suicide risk in the general population [22], differ between prisoners who had made near-lethal suicide attempts and other prisoners? Secondly, we investigated distal (e.g. childhood trauma) and more proximal (e.g. recent life events) factors, together with a range of environmental factors that related to the extent and quality of prisoners’ social networks. A deeper understanding of psychological and environmental factors will potentially contribute to understanding suicidal behaviour in prisoners, and assist in developing effective suicide prevention initiatives in prisons, and possibly in other institutional settings such as the military [23] and psychiatric inpatient units [24]. The latter settings also have elevated suicide rates, however the contribution of social and environmental factors is sometimes neglected. This more detailed understanding of suicidal behaviour in prisoners is especially important given the known difficulties in developing effective prison screening instruments [25].

## Methods

### Participating Prisons

Data were collected between 2007 and 2009 in 19 male prisons in England. These were selected in consultation with the Ministry of Justice because of high rates of suicide attempts and completed suicides (and their being within 100 miles of Oxford). Participating establishments included three Young Offenders’ Institutes (prisoners aged 18–21), three Category ‘A’ (maximum security) prisons, 12 Category B prisons (“establishments for those who do not require maximum security but for whom escape must be made difficult”) and one Category C prison (“for prisoners who cannot be housed in open conditions but who are unlikely to try to escape”).

### Participant Identification

#### Cases

Near-lethal suicide attempts were defined as acts of self-harm which a) could have been lethal had it not been for intervention or chance, and/or b) involved methods which are associated with a reasonably high chance of death [26]. These criteria have been outlined elsewhere in more detail [20]. Prison officers used these criteria to identify prisoners to refer to the study. Cases were interviewed within four weeks of the suicide attempts.

We included 60 male prisoners in the study, and excluded 42. The 60 participating prisoners were significantly more likely than those excluded to be white (52/60 (87%) vs. 25/42 (60%); χ^2^ = 9.8, p = 0.01) and to be on a life sentence (13/39 (33%) vs. 2/23 (9%); χ^2^ = 4.6, p = 0.03). There were no other significant differences between the included and excluded prisoners with regard to tested socio-demographic or criminological characteristics.

#### Controls

Prisoners who had never made a near-lethal suicide attempt whilst in prison were randomly selected by the Ministry of Justice from the Prison Service’s daily list of prisoners. As confounding of risk of suicide in prisons has been shown for age, gender, and facility type [12], controls were matched with cases in terms of age (five years older or younger), gender and type/category of prison, although each control was from a different prison to the prisoner to which they were matched. All participants were over the age of 18 years.

### Interviews

The interviews lasted between 90 and 120 minutes and took place in private in the prison. They included the following measures, some of which were abbreviated to allow for their use within the constraints of custody (Table 1):

**Table 1:** Measures used in study of near-lethal suicide attempts in male prisoners.

Characteristic	Measure	Reference
Sociodemographic	Structured questionnaire	[27]
Criminological	Structured questionnaire	[28]
Depression	Beck Depression Inventory I-A	[29]
Hopelessness	Item 2 (Hopelessness) of the Beck Depression Inventory I-A	[29]
Self-esteem	Modified version of Robson’s Self Concept Scale	[33]
Impulsivity	Plutchik Impulsivity Scale	[34], [35]
Aggression	Brown-Goodwin Assessment for Lifetime History of Aggression questionnaire	[36]
Hostility	Two subscales of the Buss-Durkee Hostility Inventory	[38]
Childhood trauma	Childhood Trauma Questionnaire	[42]
Life events and prison experiences	Structured questionnaire	[28]
Social support	Social Support Scale	[28]
Social networks	Structured questionnaire	[28]
Psychiatric diagnoses	Mini-international Neuropsychiatric Interview	[64]

#### Sociodemographic and criminological variables

Sociodemographic information was gathered using an adapted version of a structured questionnaire which has been reliably used in many previous studies of self-harm [27]. A questionnaire to collect criminological data was adapted from one used in a major study of psychiatric morbidity amongst prisoners in England and Wales [28].

#### Psychological characteristics

##### (i) Depression

The Beck Depression Inventory I-A (BDI) was used to assess levels of depression [29], [30]. This is a 21-item self-report measure where each item can score between 0 and 3. It has high levels of internal consistency, stability and validity [31].

##### (ii) Hopelessness

We used Item 2 (Hopelessness) of the BDI to assess levels of hopelessness. Beck and colleagues [32] reported that this item was almost as predictive of eventual suicide in 211 suicide ideators as the 20-item Hopelessness scale.

##### (iii) Self-esteem

We measured self-esteem using a modified version of Robson’s Self Concept Scale [33]. We modified the scoring from the original 7-point scale to a 4-point Likert scale (completely agree, agree, disagree, completely disagree). Each of the positive items scores between 1 and 4, with negative items reversed. Total scores range from 12 to 48.

##### (iv) Impulsivity

We measured impulsivity using the Plutchik Impulsivity Scale (PIS) [34], [35], which includes 15 4-point Likert scale items. Each item scores between 1 and 4, providing a total score range of 15 to 60. This scale is reported to have good validity and internal consistency [34].

##### (v) Aggression

This was measured with the Brown-Goodwin Assessment for Lifetime History of Aggression questionnaire (BGLA) [36]. We excluded two of the original nine items as they relate specifically to military issues, but retained the original scoring.

##### (vi) Hostility

This was assessed using a modified version of the Buss-Durkee Hostility Inventory (BDHI) [37], [38], [39]. As administration of the original 75-item questionnaire is time consuming, we used only two of its seven subscales: ‘assault’ (physical violence towards others) and ‘irritability’ (readiness to explode with negative affect at the slightest provocation). These two combined subscales have amongst the highest individual internal consistency coefficients of all the subscales [40], and have previously been used in studies investigating the relationship between suicide attempts and hostility [41].

#### Childhood trauma

The Childhood Trauma Questionnaire (CTQ) is a 28-item self-report inventory which assesses histories of emotional, physical and sexual abuse, and of emotional and physical neglect [42]. It has good test-retest reliability and internal consistency, and its criterion validity has been found to be acceptable [43]. However, to simplify the questionnaire, we abridged the original 5-point Likert scale to three points: often, sometimes and never, allowing a total score of between 25 and 75.

#### Life events and prison experiences

This checklist was adapted from a psychiatric morbidity survey amongst English and Welsh prisoners [28]. Each item is rated ‘yes’ or ‘no’. In addition, we included questions on whether the prisoner had ever been in local authority care, had a family history of suicide or self-harm, and whether they knew people in prison who had self-harmed or died by suicide.

#### Social support

The Social Support Scale (SSS) is a 7-item instrument measuring self-perceived social support. It has been adapted for use both in the general community [44] and in prison [28], [45]. It has three response categories: not true, partly true and certainly true. Each item is scored between 1 and 3, and overall scores range from 7 to 21. Scores under 17 indicate that participants perceive a severe lack of social support, between 18 and 20 a moderate lack of social support, and 21 indicates no lack of social support.

#### Social networks

We asked participants about their social networks in relation to extent (number of external contacts via letters, telephone calls and visits since being in prison) and quality (number of close friends and relatives that they feel close to outside and inside prison). The questionnaire was based on that used in the ONS prison study (1998).

We have elsewhere reported on associations between near-lethal self-harm in male prisoners and diagnosed psychiatric disorders [15], and on the psychosocial and psychiatric influences on female prisoner suicide [46], [47].

### Statistical Analyses

The analyses were conducted using the Statistical Package for the Social Sciences [48] and STATA [49]. We adopted a 95% (p<0.05) significance level. For continuous data, paired sample t-tests were used. Odds ratios, 95% confidence intervals and associated p-values for analyses of categorical factors were calculated using McNemar’s Test to account for matching of cases and controls.

We conducted conditional logistic regressions to determine which factors were independently predictive of near-lethal suicide attempts. Specifically, within the criminological and life events domains, we identified which variables were associated univariately with near-lethal self-harm at a 95% significance level (p<0.05), and present in at least ten pairs of participants (to avoid model instability). In addition, we dropped collinear variables to reduce the risk of over-adjustment. We analysed the sensitivity and specificity of factors that were potentially important in three separate models. These models included combinations of risk factors to examine their predictive accuracy for near-lethal self-harm. The first model included those factors that remained significant in multifactorial analyses. The second and third models added one factor each that was of borderline significance in these multifactorial analyses.

Finally, we tested associations among psychological variables, and within scores on the childhood trauma scale and subscales, using Pearson’s r (for normally distributed data) and Spearman’s rho correlations (for non-normal distributions).

### Informed Consent, Confidentiality and Ethical Approval

Prisoners who met the inclusion criteria received a participant information sheet which explained details of the study, including its purpose, what the interview would entail, and the ability of the prisoner not to participate without any adverse consequences to themselves, any medical treatment they may have been receiving, or their sentence plan, parole or any other aspect of their life in prison.

Before the interview took place, participants were reminded of the purpose of the study and what the interview would entail. Participants were asked whether they had any questions. They were assured that they could withdraw their consent for the interview at any stage without any adverse consequences. Confidentiality was assured to the prisoner, except in cases where a serious threat was posed to their, or someone else’s life. Following from these discussions, the prisoner was asked if he was willing to participate and, if so, was asked to sign a consent form.

Participants had access to support both before and after the interview from a Suicide Prevention Coordinator, Chaplain, Samaritan, Listener (trained peer support) or Psychologist.

The study had ethical approval from the UK’s Central Office for Research Ethics Committee (Ethics number 06/MRE12/83) and the Prison Service for England and Wales (Reference PG 2006 063).

Our data contains potentially identifiable information. Because of the strict confidentiality agreements in place with participants at the time of the interviews we do not intend to make our data publically available.

## Results

### Near-lethal Attempts

Two-thirds (n = 40, 67%) of the near-lethal suicide attempts were by hanging or ligaturing, 12 (20%) by severe cutting, three (5%) by self-asphyxiation, three (5%) by overdose of analgesics, one (2%) by ingestion of a foreign object and one (2%) by self-immolation.

### Sociodemographic and Criminological Variables

Sociodemographic and criminological characteristics of the cases and controls are presented in Table 2. Cases were significantly more likely than controls to be white and to have no educational qualifications. There were no differences between cases and controls in their marital or employment status prior to prison, nor in whether they had children.

**Table 2:** Sociodemographic and criminological characteristics of male prisoners who made near-lethal suicide attempts (cases) and those who had not (controls).

	Cases N = 60		Controls N = 60				
Variable	n	(%)	n	(%)	Odds Ratio (95% CI)		P-value
***Marital status*** 1							
Single (vs. married)	41	(68)	46	(77)	0.7 (0.3–1.5)		0.321
***Ethnicity***							
White	52	(87)	42	(70)			
Mixed	4	(7)	3	(5)			
South Asian	2	(3)	5	(8)			
Black	1	(2)	9	(15)			
Other	1	(2)	1	(2)			
White v. Non-white	52	(87)	42	(70)	2.7 (1.0–6.8)		0.040
***Educational Qualifications***							
None v. Any	21	(35)	11	(18)	2.4 (1.0–5.9)		0.048
***Employment status***							
Unemployed^2^ v. Employed	35	(58)	29	(48)	1.6 (0.7–3.3)		0.261
***Parent or Guardian of children***	35	(58)	31	(52)	1.3 (0.6–2.8)		0.451
***Prior prison sentence***	54	(90)	40	(67)	4.5 (1.5–13.3)		0.007
***Number of prior prison spells***							
2 or more v. 0 or 1	43	(72)	31	(52)	2.1 (1.0–4.3)		0.044
***Index Offence***							
Violence	16	(27)	13	(22)			
Sexual	7	(12)	14	(23)			
Robbery	13	(22)	6	(10)			
Burglary	12	(20)	11	(18)			
Other theft	4	(7)	3	(5)			
Drugs	3	(5)	5	(8)			
Other^3^	5	(8)	8	(13)			
Violent^4^ v. Non-violent	36	(60)	33	(55)	1.3 (0.6–2.8)		0.549
***Status***							
Remand	21	(35)	12	(20)	2.3 (0.9–5.6)		0.068
Sentenced	39	(65)	48	(80)			
***Sentence type*** 5							
Life	13/39	(33)	16/48	(33)			
Determinate sentence	26/39	(67)	32/48	(67)			
Less than or equal to 6 months	3/26	(12)	0/32	(0)			
Greater than 6 months to less than a year	1/26	(4)	2/32	(6)			
12 months to less than 4 years	14/26	(54)	17/32	(53)			
4+ years	8/26	(31)	13/32	(41)			
***Latency***							
Less than 30 days since 1^st^ reception	17	(28)	1	(2)	17.0 (2.3–127)		0.006
Less than 30 days in current prison	25	(42)	1	(2)	25.0 (3.4–185)		0.002
***Single cell*** 6	30/59	(51)	29	(48)	1.1 (0.5–2.5)		0.715
**On current sentence been:**							
Held in solitary confinement	23	(38)	14	(23)	2.0 (0.9–4.5)		0.090
Held in a ‘safer cell’ 6,7	17/55	(31)	3	(5)	?^2^ = 14.2		<0.001
Given ‘added days’ for disciplinary offences	7	(12)	6	(10)	1.2 (0.4–3.5)		0.782

1 Single includes being divorced, separated or widowed; Married includes having a partner.

2 Unemployed includes sick/disabled.

3 Including criminal damage, fraud and forgery.

4 Including violence, sexual and robbery.

5 Applies to sentenced prisoners only.

6 For cases at incident; for controls at interview.

7 Odds ratio undefined when there is a 0 in one or more cells (McNemar’s chi-square and associated p-value reported where possible when observed values are equal to or greater than 10).

Cases were significantly more likely than controls to have had a prior prison sentence, and to have had two or more (compared to none or only one) prior prison sentences. Cases were significantly younger when they received their first conviction (mean age in years = 15.8, SD = 5.4, range = 10–28 v. 17.9, SD = 6, range = 10–28; p = 0.034). Whilst there was no statistical difference between cases and controls in terms of their prison status (remand or sentenced), cases were significantly more likely to have been in prison for less than 30 days since first reception and for less than 30 days in the current prison.

There was no significant difference between cases and controls with respect to their category of index offence. In particular, cases were no more likely than controls to have been arrested or convicted of a violent act. Whilst it was not possible, due to lack of statistical power, to determine whether there was a difference in sentence type (life or determinate) between cases and controls, the data do not suggest any strong differences: 13 (33%) of 39 cases who had been sentenced were serving a life sentence compared to 16 (33%) of 48 controls who were also on life sentences. Similarly, for those prisoners serving a determinate sentence, there were no differences in the length of sentences between cases and controls (Table 2).

Whilst in prison for the current offence, cases were significantly more likely than controls to have been held in a ‘safer cell’ (cells with reduced ligature points and often with a clear Perspex door to facilitate observation). Cases were also significantly less likely to have been employed in prison. There were no significant differences between cases and controls in whether they had been held in solitary confinement (for disciplinary reasons), given ‘added days’ for disciplinary offences, or taken part in drug or alcohol misuse or education programmes on their current sentence.

No multifactorial analysis was conducted as none of the possible factors met the inclusion criteria of univariate significance and statistical stability except having had a prior prison sentence.

### Psychological Characteristics

Compared to controls, cases had significantly more depressive symptoms, higher hopelessness, impulsivity, aggression and hostility scores, and significantly lower self-esteem scores (Table 3). All psychological variables were significantly intercorrelated (Table 4).

**Table 3:** Psychological characteristics and reported childhood trauma of male prisoners who made near-lethal suicide attempts (cases) and those who had not (controls).

	Cases N = 60	Controls N = 60	
	Mean (SD)/Median	Mean (SD)/Median	Paired Sample T-test/Wilcoxon Signed Ranks Test
Depression^1^	31.8 (12.6)	13.9 (10.5)	t = 8.2, df = 59, p<0.001
Hopelessness^2^	2^1^	1^1^	z = −4.7, p<0.001
Self-esteem^3^	28.5 (5.9)	35.3 (5.2)	t = −7.1, df = 59, p<0.001
Impulsivity^4^	37.8 (8.2)	31.2 (7.4)	t = 4.6, df = 59, p<0.001
Aggression^5^	18.9 (6.7)	13.5 (7.5)	t = 3.9, df = 59, p<0.001
Hostility^6^	11.4 (5.2)	8.8 (4.2)	t = 2.9, df = 59, p = 0.01
Childhood trauma^7^	39.3 (11.2)	33.9 (9.9)	t = 3.1, df = 59, p = 0.01
Emotional abuse^8^	8.7 (3.3)	7.1 (2.6)	t = 3.2, df = 59, p = 0.01
Physical abuse^8^	6	5	z = −1.87, p = 0.06
Sexual abuse^8^	5	5	z = −0.52, p = 0.60
Emotional neglect^8^	9.2 (3.1)	7.5 (2.7)	t = 3.2, df = 59, p = 0.01
Physical neglect 8	7	5	z = −2.62, p = 0.01

1 Scores can range from 0 to 63, with higher scores indicating greater levels of depression.

2 Scores can range from 0 to 3, with higher scores indicating greater levels of hopelessness.

3 Scores can range from 12 to 48, with higher scores indicating greater levels of self-esteem.

4 Scores can range from 15 to 60, with higher scores indicating greater levels of impulsivity.

5 Scores can range from 0 to 28, with higher scores indicating greater levels of aggression.

6 Scores can range from 0 to 21, with higher scores indicating greater levels of hostility.

7 Scores could range from 25 to 75, with higher scores indicating greater levels of trauma.

8 Scores could range from 5 to 15, with higher scores indicating greater levels of trauma.

**Table 4:** Correlation matrix of impulsivity, hostility, self-esteem, aggression and depression scores in all prisoners participating in the study (N = 120).

	Impulsivity	Hostility	Self-esteem	Aggression	Depression
Impulsivity	1.00				
Hostility	0.56* b	1.00			
Self-esteem	−0.57* b	−0.50* b	1.00		
Aggression	0.56* a	0.65* a	−0.40*a	1.00	
Depression	0.56* a	0.47* a	−0.74*a	0.34* a	1.00

* p<0.0001 for correlations and case-control comparisons.

a Correlation coefficient calculated using Spearman’s rho.

b Correlation coefficient calculated using Pearson’s r.

### Childhood Trauma

Cases scored significantly higher than controls on the Childhood Trauma Questionnaire, and on three of its subscales: emotional abuse, emotional neglect, and physical neglect (Table 3). Scores on all subscales were significantly intercorrelated at p<0.001 (Table 5).

**Table 5:** Correlation matrix of scores on the childhood trauma scale and subscales in all prisoners participating in the study (N = 120).

	Childhood trauma	Sexual abuse	Emotional abuse	Physical abuse	Emotional neglect	Physical neglect
	1.00					
Sexual abuse	0.42*	1.00				
Emotional abuse	0.85**	0.29**	1.00			
Physical abuse	0.78**	0.33**	0.64**	1.00		
Emotional neglect	0.87**	0.22*	0.66**	0.55**	1.00	
Physical neglect	0.83**	0.28**	0.63**	0.51**	0.73**	1.00

* p<0.05.

** p<0.01.

All correlation coefficients were calculated using Spearman’s rho.

Approximately 40% of cases reported having been emotionally (n = 25, 42% *vs.* n = 16, 27% in controls; OR = 2.0, 95% CI 0.8–5.1, p = 0.08) or physically (n = 25, 42% *vs.* n = 12, 20%; OR = 3.6, 95% CI 1.3–12.4, p = 0.01) abused as children. There was no statistically significant difference between the numbers of cases and controls who reported having been sexually abused as children (n = 7, 12% *vs.* n = 5, 8%; OR = 1.5, 95% CI 0.4–7.2, p = 0.53).

### Life Events

Most life events were reported more frequently by cases than controls (Table 6). The association between having experienced adverse life events and a near-lethal act was significant for bullying, having been homeless, and having experienced the death of a parent or sibling. Cases were also significantly more likely than controls to have been in local authority care under the age of 16 years. Cases had also experienced significantly more types of life events than controls (n = 6.5, SD = 3.0, range = 1–13 *vs.* n = 5.1, SD = 3.0, range = 0–12; p = 0.01). Finally, significantly more cases than controls had experienced their most recent life event in the last year and there was a trend towards more having experienced it in the last 6 months.

**Table 6:** Life events, experiences of victimization in prison, and exposure to suicide and self-harm of male prisoners who made near-lethal suicide attempts (cases) and those who had not (controls).

	Cases N = 60		Controls N = 60			
	N	(%)	n	(%)	Odds Ratio (95% CI)	P-value
***Have you ever experienced any of the following*** ***problems or events:***						
Bullying	37	(62)	20	(33)	3.4 (1.5–8.0)	0.01
Violence at work^1^	3	(5)	5	(8)		
Violence in the home	24	(40)	14	(23)	2.3 (1.0–5.2)	0.06
Sexual abuse	11	(18)	5	(8)	3.0 (0.8–11.1)	0.10
Serious/life-threatening illness/injury	22	(37)	18	(30)	1.3 (0.6–2.6)	0.48
Separation due to marital difficulties orthe breakdown of a relationship	30	(50)	35	(58)	0.6 (0.3–1.5)	0.30
Death of husband/wife (or partner) or child^1^	8	(13)	5	(8)		
Death of a parent or brother/sister	27	(45)	16	(27)	2.2 (1.0–4.9)	0.05
Death of a close family friend or otherrelative you were close to	41	(68)	38	(63)	1.2 (0.6–2.3)	0.61
Stillbirth of a baby^1^	6	(10)	3	(5)		
Expelled from school	39	(65)	36	(60)	1.2 (0.6–2.5)	0.59
Sacked or made redundant	19	(32)	25	(42)	0.7 (0.3–1.4)	0.28
Run away from home	35	(58)	29	(48)	1.5 (0.7–3.1)	0.28
Been homeless	35	(58)	20	(33)	3.1 (1.3–7.4)	0.01
Serious money problems	27	(45)	22	(37)	1.4 (0.7–2.8)	0.37
Local authority care	26	(43)	12	(20)	3.0 (1.3–7.1)	0.01
Multiple v. One Placement^1^	13	(22)	5	(8)		
***Date of most recent life event***						
In the previous 6 months	24	(40)	15	(25)	1.8 (0.9–3.8)	0.11
In the previous year	33	(55)	18	(30)	2.7 (1.2–5.7)	0.01
***In prison for this current offence have you:***						
Been threatened with violence	25	(42)	16	(27)	2.1 (0.9–4.9)	0.08
Been the victim of actual abuse	14	(23)	7	(12)	2.4 (0.9–6.8)	0.10
Had any of your belongings stolen	20	(33)	13	(22)	2.0 (0.1–5.0)	0.13
Been intimidated to hand over any of your belongings (‘taxed’)^1^	6	(10)	2	(3)		
Received unwanted sexual attention^1^	3	(5)	1	(2)		
Been the victim of forced sexual attentions^1^	0	(0)	0	(0)		
***Have you ever experienced?***						
Family self-harm 2,3	23/53	(43)	15/56	(27)	2.1 (0.9–5.7)	0.07
Family died by suicide^3^	9/53	(17)	4/56	(7)	3.0 (0.7–17.2)	0.08
Family self-harm^2^ and/or died by suicide^3^	27/53	(59)	18/56	(32)	2.6 (1.0–7.3)	0.03
Friends self-harm^1, 2^	5/51	(10)	7/56	(13)		
Friends died by suicide^3^	12/53	(23)	17/56	(30)	0.9 (0.3–2.5)	0.82
Knew of self-harm or suicide in prison^3^	37/52	(71)	39/57	(68)	1.0 (0.4–2.7)	1.00

1 Odds ratios not calculated for disorders where number of discordant pairs was less than 10.

2 Includes self-harm without suicidal intent and attempted suicide.

3 Due to missing data, analysis conducted with 49 pairs.

When having been bullied, homeless, and having a history of being in Local Authority care were entered into a multifactorial regression model, only bullying (OR = 2.5, 95% CI 1.0–6.3, p = 0.04) remained significant.

More cases than controls had experienced non-fatal deliberate self-harm or death by suicide in biological family members and when these phenomena were combined the difference was significant (Table 6). There was no difference between cases and controls in terms of having friends who had deliberately self-harmed or died by suicide. Cases were no more likely than controls to have been exposed to self-harm or suicidal behaviours whilst in prison.

### Experiences in Prison

Almost all experiences of victimization in prison (apart from sexual abuse where no cases or controls reported being a victim) were reported more frequently by cases than controls, although these differences did not reach statistical significance (Table 6). Compared to controls however, cases had experienced significantly more types of victimization in prison (n = 1.1, SD = 1.3, range = 0–4 *vs*. n = 0.7, SD = 1.0, range = 0–4; p = 0.01).

### Social Support

Prisoners making a near-lethal suicide attempt reported lower levels of self-perceived social support than controls on the Social Support Scale (mean = 16.6, SD = 3.7, range = 8–21 *vs*. mean = 18.9, SD = 2.4, range = 11–21; p<0.001).

### Social Networks

Cases were significantly more likely than controls to report none or few close or good friends outside prison, and controls to report having no close or good friends living or working inside prison (Table 7).

**Table 7:** Social networks and external contacts of male prisoners who made near-lethal suicide attempts (cases) and those who had not (controls).

	Cases N = 60		Controls N = 60			
Variable	n	(%)	n	(%)	Odds Ratio (95% CI)	P-value
***Social Networks:***						
***Relatives you feel close to:***						
None	11	(18)	4	(7)		
One	10	(17)	3	(5)		
Between 2 and 5	26	(43)	31	(52)		
More than 5	13	(22)	22	(37)		
None v. Any	11	(18)	4	(7)	3.3 (0.9–12.1)	0.067
***Number of close or good friends outside prison:***						
None	23	(38)	8	(13)		
One	10	(17)	8	(13)		
Between 2 and 5	20	(33)	23	(38)		
More than 5	7	(12)	21	(35)		
None v. Any	23	(38)	8	(13)	4.0 (1.5–10.7)	0.006
***Visit from or speak with close or good friends or*** ***relatives outside prison in past 7 days***	22	(37)	33	(55)	0.5 (0.2–1.0)	0.061
***Number of close or good friends who live or work*** ***inside prison:***						
None	30	(50)	15	(25)		
One	10	(17)	10	(17)		
Between 2 and 5	13	(22)	22	(37)		
More than 5	7	(12)	13	(22)		
None v. Any	30	(50)	15	(25)	2.7 (1.2–5.7)	0.012
***External Contacts:***						
***Letters from family/friends*** 1	50	(83)	59	(98)		
0–2 persons sent letters	34	(57)	13	(22)		
3–5 persons sent letters	23	(38)	33	(55)		
6–8 persons sent letters	3	(5)	14	(23)		
***Phone calls from family/friends***	51	(85)	58	(97)	0.1 (0.01–1.0)	0.05
0–2 persons made phone calls	36	(60)	14	(23)		
3–5 persons made phone calls	23	(38)	37	(62)		
6–8 persons made phone calls	1	(2)	9	(15)		
***Visits from family/friends***	42	(70)	50	(83)	0.4 (0.2–1.1)	0.082
0–2 persons visited	44	(73)	27	(46)		
3–5 persons visited	15	(25)	20	(34)		
6–8 persons visited	1	(2)	12	(20)		

1 Odds ratios not calculated for disorders where number of discordant pairs was less than 10.

### Sensitivity and Specificity of Possible Models

We analysed the sensitivity and specificity of factors that were potentially important in three different models. The first included those factors that remained significant in multifactorial analyses (i.e. having had a prior prison sentence and having been bullied). Both factors were present in 34 cases and 13 controls (23 cases and 34 controls had only one of these factors; 3 cases and 13 controls had none). The model’s sensitivity was 0.57 and specificity was 0.78. In other words, using these factors to predict near-lethal self-harm in prisoners means that one would correctly identify 57% of cases, and also correctly identify 78% who were not at risk.

The second model included being white, having had a prior prison sentence, and having been bullied. All three factors were present in 30 cases and 9 controls. The model’s sensitivity was 0.50, its specificity was 0.85.

The third model included being white, having a family history of self-harm or suicide, a prior prison sentence, and having been bullied. All four factors were present in 14 cases and 4 controls. The model’s sensitivity was 0.23 and specificity was 0.90. Thus, as expected, the sensitivity of the model was reduced by adding to the numbers of factors in any predictive model, but the specificity increased.

There was no improvement in these models when physical abuse was added or when it substituted another factor.

## Discussion

We interviewed 60 men who made near-lethal suicide attempts in prison and 60 prisoners who had never made a near-lethal suicide attempt whilst incarcerated using a semi-structured interview covering a wide range of psychological and environmental factors. We found that a number of psychological characteristics, and factors measuring childhood trauma, life events, social support and social networks, significantly differentiated prisoners who made near-lethal suicide attempts from those who did not. Below we consider these findings in more detail, before bringing them together with our previously reported findings for psychiatric disorder and history of previous self-harm [15] into an explanatory model for near-lethal suicidal behaviour in prisoners, which we argue is applicable to completed suicide [21].

Our findings have different implications depending on the comparison group. Compared with the first comparison group, prisoners who did not make near-lethal suicide attempts, our data provide novel information on psychological and environmental risk factors for suicidal behaviour in prison. This has implications for suicide prevention strategies in prison and developing a model of near-lethal self-harm in prisoners. An alternative comparison is with the general population, and examining the findings in this way provides some insight into suicidal behaviour in prison.

Compared with controls, cases had significantly higher levels of depressive symptoms, hopelessness, impulsivity, aggression, hostility, and lower levels of self-esteem. These results are in keeping with previous research on the role of personality characteristics in suicidal behaviour, both in prison [50], [51], [52], [53] and in the community [6], [18].

Prisoners who undertook near-lethal suicide attempts were also more likely than controls to have experienced childhood trauma, especially emotional or physical abuse, or emotional neglect, as has previously been reported in the community [54]. Similarly, suicidal ideation and attempts were significantly associated with childhood trauma in studies of male prisoners in Italy [51], England and Wales [55], and in female prisoners in the US [56]. However, in this report, the difference between cases and controls in self-reported rates of child sexual abuse did not reach statistical significance, in contrast to our parallel study of female prisoners [47] and other previous research [57]. This apparent difference might be because previous studies have tended to use either mixed-sex or women-only samples of inmates, which have a higher prevalence of child sexual abuse [58]. It is possible that child sexual abuse amongst men is not as important a risk factor for suicide in prison as it is for women. Alternatively, it might be that the method of data collection through face-to-face interview dissuaded male prisoners from admitting to sexual abuse in childhood.

Adverse life events were common amongst the prisoners in this study. However, because of the high rates of these life events in the control group, a finding consistent with previous research [57], [59], the only significant differences between cases and controls were for bullying, homelessness, death of a parent or sibling, and having been in Local Authority care. Of these, bullying remained significant in multivariate analysis. It is possible that more modest associations with near-lethal suicide attempts may have become apparent with a larger sample because the prevalence of specific life events was generally higher in cases than in controls. Also, there may have been differences in their degree or impact, rather than simply their presence or absence.

Cases had experienced significantly more types of life events than controls. Similarly, the ONS prison study [19] found that nearly three times as many male prisoners in England who had tried to kill themselves in the previous year had experienced seven or more events than those who had never attempted suicide. Thus there may be a cumulative effect of life events on the likelihood of attempting suicide, i.e. it is not only the presence or absence of any life event but the number and impact of life events (possibly within a given amount of time) experienced by an individual. It may also be that the relationship between number of life events and risk of suicide is not linear, but there may exist a ‘tipping point’ at which an individual’s ability to cope with adverse events is breached.

More cases than controls had experienced a family member either self-harming (with or without suicidal intent) or dying by suicide. This is consistent with both genetic [60] and social learning models of suicidal behaviour [61]. However, exposure to self-harm or suicide in friends or fellow prisoners did not differ between cases and controls, the latter finding probably reflecting the high incidence of self-harm and attempted suicide in prisons [55].

Poor social support was associated with making a near-lethal suicide attempt in prison. Specifically, we found the following factors were associated with such attempts at least at borderline significance levels: having fewer close or good friends both outside and inside prison, feeling less close to relatives, and having fewer external contacts in the form of letters, phone calls and visits. These findings are consistent with previous prison and community studies [62], [63] and suggest that a person’s social environment can contribute to suicide risk. It may be that the more an individual feels connected to social surroundings, the more likely they are to be socialised into the norms of the group or society and consequently less likely to pursue self-harming behaviours and suicide. In prison, this feeling of connectedness may be even more important than in the community since incarceration has already removed the individual from their primary support group in most prisoners. Previous research [9], [10] suggests that other institutional and environmental factors may have an impact on prisoners’ feelings of connectedness, and potentially on risk of near-lethal self-harm. However, as we matched participants by type of establishment, we were not able to fully test these hypotheses.

The risk factors identified above are similar to findings from general population studies, where individuals who die from suicide or engage in suicidal behaviour are also more likely to have suffered childhood trauma or other adverse life events, [54], have a family member engaged in suicidal behaviour [60], [61], have poor social support networks [62], [63], and also have higher levels of depressive symptoms, hopelessness, impulsivity, aggression, hostility, and lower levels of self-esteem [6], [18]. Whilst the risk factors presented in this report appear similar compared to the general population, the background prevalence of these risk factors is much higher in prisoners than in the general population and therefore any interventions focusing on them might have the potential for a greater impact.

### Towards a Comprehensive Model of Near-lethal Self-harm in Prisoners

**Figure 1:**
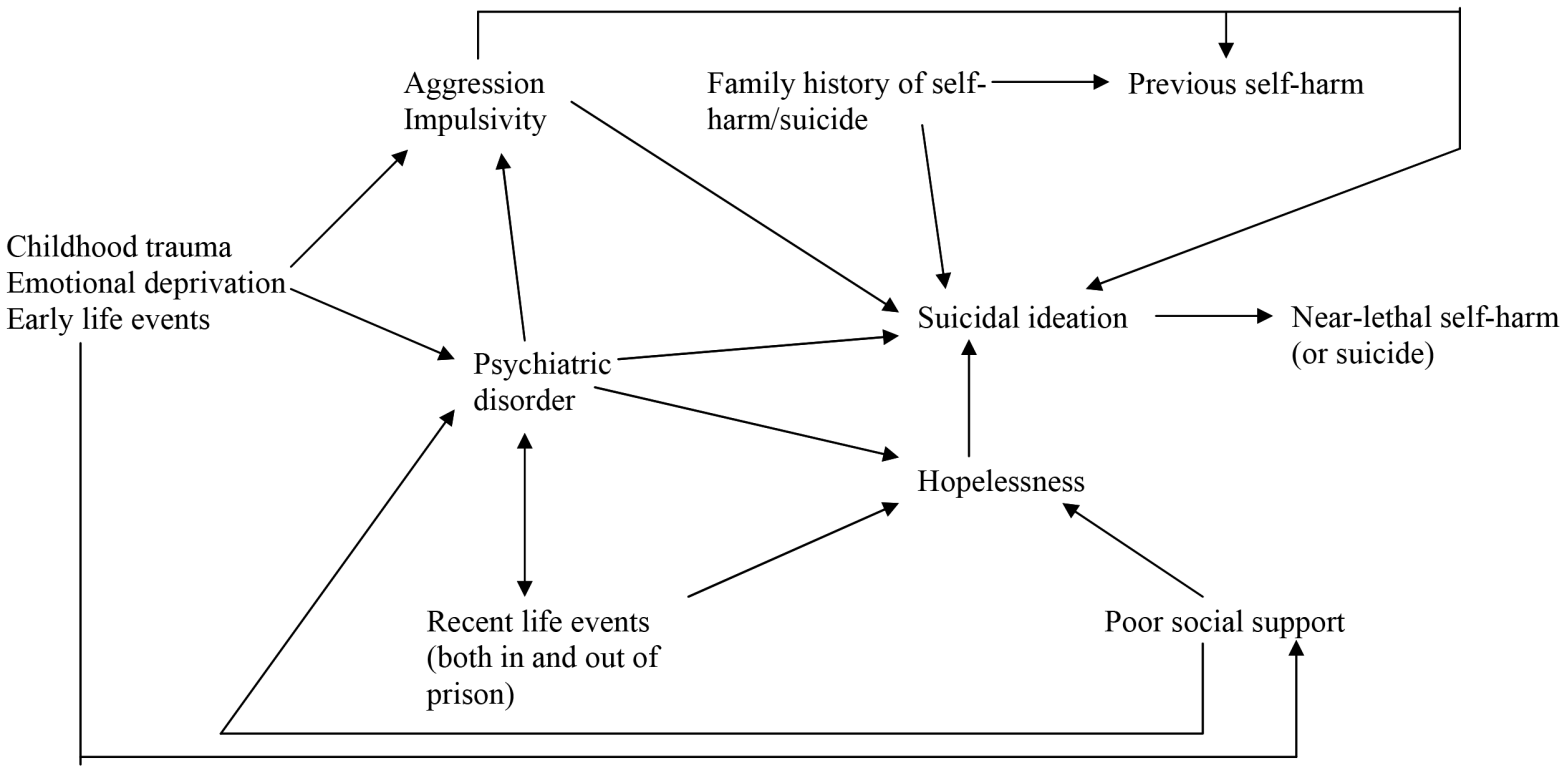
A model of possible pathways for suicide in prisoners.

The findings of this study, together with previously published data about the role of psychiatric disorders in prisoners’ near-lethal self-harm [15], provide support for a theory of prison suicide which incorporates both historical (or lifetime) factors that may make a person vulnerable to suicide as well as prison-related ones, and clinical factors. There are likely to be complex interactions between these factors (Figure 1).

The multifactorial analyses we conducted suggest that prior prison spells and having being bullied are independent factors for near-lethal self-harm in prisoners, possibly alongside white ethnicity and having a family history of suicide and self-harm. Our predictive models were, however, limited by low sensitivity (i.e. high false negative rate), which is likely to be partly a consequence of the models not including any psychiatric diagnostic variables and, possibly, ecological factors such as overcrowding [10]. Adding more factors to these models decreased sensitivity further, highlighting the problem of predicting rare events using risk factors that are common in the population of interest. Nevertheless, these models may be improved, and could be useful in settings where identification of high risk is problematic. Further research is currently underway to determine whether adding psychiatric variables to this model could improve its predictive power.

### Strengths and Limitations of the Study

Studying survivors of near-lethal self-harm appears to provide a good proxy for completed suicide in prison [21], and allows assessment of a wide range of potentially contributing factors [20]. However, due to time limitations, we measured some of these variables using shortened or simplified versions of existing questionnaires. Whilst most of the altered versions of the questionnaires were psychometrically validated, some (e.g. the Robson Self-Concept Scale) were not. Also, all of the measures used relied on prisoners’ self-report.

Interviews were conducted up to four weeks after the act. However, as some of the risk factors that were investigated were trait measures, these should largely have been unaffected by the time delay. Also, whilst the study was adequately powered to test for associations with the main variables, it was underpowered to test for more modest associations. It should be emphasized, however, that conducting a study like this is complex and very laborious and therefore restricted sample size is an inevitable consequence. As we tested multiple associations, it is possible that some of the significant findings at the 5% level were chance findings. Therefore caution is warranted in some of the less strong findings, and these will need replication. Nevertheless, our findings highlight the co-occurrence of criminological and psychosocial problems associated with the risk of suicide, and provide a useful basis for future research.

### Conclusions

There are high absolute and relative rates of suicide and self-harming behaviours in prisons in many countries, and prisoners have been identified as a high risk group in national suicide prevention strategies. The findings of this study have implications for suicide prevention both in prisons in the UK and elsewhere, and also perhaps for other institutionalised populations. They support a model of suicidal behaviour in prisons that incorporates both imported risk factors (i.e. characteristics and experiences that individuals already have at the time of entry to prison) and environmental risk factors, including influences in the prison setting. This suggests assessment of suicide risk at the time of reception into prison could include any history of suicidal behaviour in the family, childhood trauma, previous self-harm, adequacy of social networks, mental health, and levels of self-esteem, aggression, and impulsivity. Those who experience adverse life events while in prison, especially if their social support appears to be restricted, are at heightened risk, and careful monitoring of such individuals who have concurrent risk factors should be considered.
